# Genetic Diversity and Population Structure of Two Mexican Creole Cattle Populations

**DOI:** 10.3390/ani16101450

**Published:** 2026-05-09

**Authors:** Néstor Gerardo Michel-Regalado, Clemente Lemus-Flores, Miguel Ángel Ayala-Valdovinos, Fernando Grageola-Núñez, Víctor Hugo Severino-Lendechy, Theodor Duifhuis-Rivera, Guillermo Martínez-Velázquez, Gilberto Lemus-Ávalos, Jorge Barzilai Lara-Castillo

**Affiliations:** 1 Posgrado en Ciencias Biológico Agropecuarias, Universidad Autónoma de Nayarit, Tepic 63315, Mexico; nestor.michel@academicos.udg.mx (N.G.M.-R.); lemus.ag91@gmail.com (G.L.-Á.); 22000023@uan.edu.mx (J.B.L.-C.); 2 Centro Universitario de Ciencias Biológicas y Agropecuarias, Departamento de Producción Animal, División de Ciencias Veterinarias, Universidad de Guadalajara, Zapopan 45101, Mexico; theodor.duifhuis@academicos.udg.mx; 3 Unidad Académica de Medicina Veterinaria y Zootecnia, Universidad Autónoma de Nayarit, Tepic 63315, Mexico; clemus@uan.edu.mx (C.L.-F.); fgrageola@uan.edu.mx (F.G.-N.); 4 Centro de Estudios Etnoagropecuarios, Universidad Autónoma de Chiapas, San Cristóbal de las Casas, Chiapas 29264, Mexico; vhseverino@hotmail.com; 5 Campo Experimental Santiago Ixcuintla INIFAP-CIRPAC, Santiago Ixcuintla 63300, Nayarit, Mexico; gmv1us@yahoo.com

**Keywords:** Creole cattle, Coreño, Raramuri, SNP, *Bos taurus*

## Abstract

The descendants of the first cattle that arrived in America in 1493 are known as Creole cattle. The objective of this study was to characterize the diversity and genetic structure of two Creole cattle populations in Mexico. A total of 75 cattle were included: 36 Coreño (CC) and 39 Raramuri (CR). Genotyping was performed using an SNP microarray. Quality control, estimation of genetic diversity, and analysis of homozygosity by runs of homozygosity (ROH) and linkage disequilibrium (LD) analyses were performed in PLINK v1.9. The effective population size (*N_e_*) was calculated using GONE2 and CurrentNe2. The structure was analyzed by principal component analysis (PCA), discriminant analysis of principal components (DAPC) and ADMIXTURE. The expected heterozygosity was high in both populations (CC 0.379 and CR 0.398). The short segments of classes > 0.5–4 represented 84.41% in the CC cattle and 92.83% in the CR cattle. The contemporary *N_e_* was 32 in the CC cattle and 473 in the CR cattle. The Creole cattle were closely grouped with European cattle populations. The ADMIXTURE revealed 70.60% of a Creole ancestral component in the CR cattle, whereas the CC cattle shared 38.67% of this component with the CR cattle and exhibited 46.44% of a differentiated Creole component. In conclusion, the two Mexican Creole cattle populations had high genetic diversity and low homozygosity; a greater extension of LD was observed for the CC cattle, and consequently, the *N_e_* was lower. The CC cattle exhibited shared ancestry with the CR cattle.

## Simple Summary

Creole cattle are considered valuable animal genetic resources because of the genetic variation associated with the adaptations they have accumulated over generations. The objective of this study was to analyze two populations of Creole cattle (Coreño and Raramuri) in Mexico using genome-wide single-nucleotide polymorphisms (SNPs). To genetically characterize these populations, the diversity among the Creole cattle was studied, and their population structure was determined with respect to reference populations. The genetic diversity of the Creole populations was high, whereas homozygosity was low and was associated with ancient historical events. The Creole populations of Mexico clustered close to the European populations and shared their own Creole ancestral component. These findings provide crucial genetic information for the establishment of conservation programs for these populations, particularly for Coreño, where a relatively small contemporary effective size was found.

## 1. Introduction

In the historical colonial context, the term “Creole” was used to distinguish people who descended from European settlers born in America, whereas in livestock studies, the term refers to populations derived from animals introduced from the Iberian Peninsula, which were established locally and adapted to different regional environments in America [1]. Creole cattle are recognized as the descendants of the first cattle that arrived on the continent in 1493 [2]. They were later introduced into what is now Mexico in the early colonial period in 1521 through the coasts of the Gulf [3]. After their introduction, Creole cattle spread rapidly across the continent with little or no human intervention; thus, through a process of natural selection, they developed adaptations to challenging local conditions [4]. In Mexico, several Creole cattle populations adapted to contrasting regional environments have been identified, including Coreño (CC) and Raramuri (CR) cattle [5]. Coreño cattle are linked to traditional production systems in the Nayar area of the Sierra Madre Occidental of Nayarit, where they have persisted under environmentally heterogeneous and seasonally variable conditions [6,7,8]. In contrast, Raramuri cattle are associated with indigenous production systems in the Sierra Tarahumara of Chihuahua, a region characterized by marked climatic variation, limited forage availability, and relative geographic isolation, factors that may have contributed to the preservation of their genetic background [4,9]. Recently, the dissemination of exotic germplasm selected for high meat or milk productivity has increased the risk of losing the genetic diversity of these populations, which have exhibited local adaptations for more than 500 years [10]. SNP microarray studies have revealed substantial genetic diversity and population structure in several Creole cattle populations from Mexico and the United States [4,11,12]. However, genomic information remains limited for some Mexican Creole cattle populations, restricting a clearer understanding of their current diversity, homozygosity patterns, linkage disequilibrium, population structure and demographic status. As a result, the current genomic status and conservation priorities of populations such as Coreño and Raramuri cattle remain insufficiently characterized. Such information is essential to support conservation and management decisions for local animal genetic resources, as has been done for other Creole species in Mexico [13,14]. Therefore, the aim of this study was to characterize the genetic diversity, homozygosity patterns, linkage disequilibrium, contemporary effective population size, and population structure of two Mexican Creole populations using SNP microarray data, in order to generate information relevant for their conservation and management.

## 2. Materials and Methods

### 2.1. Sampling

The study was approved under the name “Study of genetic diversity and population structure of Creole cattle of Mexico” by the Secretariat of Research and Postgraduate of the Autonomous University of Nayarit (registration number SIP25057). A total of 75 Creole cattle (36 CC females and 39 CR females) belonging to two populations located west (Nayarit, Lat. 22°13′09.6″ N and Long. 104°39′06.4″ W) and south (Chiapas, Lat. 17°11′00″ N and Long. 93°00′00″ W) of Mexico, respectively, were selected on the basis of the phenotypic traits described for the biotype of Mexican Creole cattle [15]. Information on the origin of the cows was considered to reduce any relatedness between the cattle. Blood samples from coccygeal vein punctures were collected from the Creole cattle in Vacutainer tubes with ethylenediaminetetraacetic acid (EDTA) (Becton Dickinson, Franklin Lakes, NJ, USA) in accordance with the provisions of NOM-051-ZOO-1995 on the humane treatment of animals during their mobilization and the guide for the care and use of agricultural animals in research and teaching [16]. Blood samples were placed on collection cards to extract the deoxyribonucleic acid (DNA).

### 2.2. Genotyping and Quality Control

The samples were genotyped using the GGP bovine 100K GeneSeek Genomic Profiler Bovine SNP microarray (Neogen, Lansing, MI, USA), which contains 95,256 SNPs mapped according to the ARS-UCD 1.2 assembly. SNP quality control was performed in PLINK v1.9 [17] by retaining markers with a call rate > 0.98, individuals with a call rate > 0.98 and SNPs with a minor allele frequency (MAF) > 0.01. Only autosomal SNPs (BTA1–BTA29) were retained for further analyses.

### 2.3. Genetic Diversity

The minor allele frequency (MAF), observed heterozygosity (Ho), expected heterozygosity (He), fixation index (Fis) and inbreeding coefficient (F) were calculated using the commands --het and --freq in the PLINK v1.9 software [17]. To evaluate the degree of genetic differentiation between the two Creole cattle populations, the Fst index was calculated using the --fst commands in PLINK v1.9 software [17]. The estimation of homozygosity was performed via the runs of homozygosity (ROH) approach through the sliding window method using --homozyg in PLINK v1.9 [17]. To capture short segments representative of ancient ancestry, a minimum length of 500 kb (--homozyg-kb) was established; to reduce the probability of detecting false-positive segments, a minimum of 36 SNPs per run (--homozyg-snp) was established considering an α of 0.05 in accordance with the methodology of Lencz et al. [18] and adapted by Purfield et al. [19]; to reduce the detection of artificial windows, a maximum bridge of 1 Mb was considered (--homozyg-gap); a minimum density of 1 marker every 50 kb (--homozyg density) was allowed, resulting in 1 missing SNP (--homozyg-window-missing 1) and 1 missing heterozygous genotype (--homozyg-het). The number of ROH segments for each individual was calculated and divided into the following categories: >0.5–1 Mb, >1–2 Mb, >2–4 Mb, >4–8 Mb, >8–16 Mb and >16 Mb. The genomic inbreeding coefficient based on ROH (FROH) was calculated for each individual using the formula proposed by McQuillan et al. [20]: FROH = ∑LROH/LAUTO, where LROH represents the total cumulative length of the runs of homozygosity detected in each individual, and LAUTO represents the total length of the autosomal genome (2489.39 Mb), corresponding to the size of the autosomes (BTA1–BTA29) in the bovine ARS-UCD 1.2 reference assembly. Population-level estimates were obtained by summarizing each parameter according to its underlying analytical scale, either across SNPs or across individuals. MAF was estimated separately for CC and CR and summarized as the mean ± standard deviation (SD) of per-SNP MAF values within each population. Ho, He, Fis, F, and FROH were derived from individual-level estimates and summarized within each population as mean ± SD.

### 2.4. Linkage Disequilibrium and Effective Population Size (N_e_)

To estimate the decay of linkage disequilibrium (LD), the LD was quantified as the squared correlation coefficient (r^2^) between pairs of SNPs in each autosomal chromosome (BTA1–BTA29). The values of r^2^ were calculated with PLINK v1.9 [17] using the --r2 command and considering all pairs of SNPs within a physical window of 0–1 Mb, retaining the complete set of comparisons with the parameters --ld-window 99999, --ld-window-kb 1000 and --ld-window-r2 0. To construct the decay curve, the SNP pairs were grouped by physical distance in 1 kb bins. The average r^2^ in each bin was estimated, and r^2^ was plotted as a function of distance. The extent of LD decay was summarized as the distance at which r^2^ dropped below 0.20, which was used as a reference threshold to compare LD decay between populations under the same analytical framework and to allow comparison with previous cattle studies that applied the same cutoff. Additionally, the average r^2^ per chromosome was calculated to evaluate the heterogeneity of the LD between the autosomes. To determine the consistency between the value of r^2^ and the chromosomal profile of the populations, a Spearman rank correlation test was performed, and its significance was evaluated by a permutation test with 100,000 permutations randomly reassigning the values of chromosomal r^2^ in the CR cattle. With the same permutation scheme, the overlap of shared extreme chromosomes was evaluated, defined as the top/bottom 25% of the average r^2^ within each population, and the analysis was performed in R software v4.3.1 [21]. The visualizations were performed in R [21] using ggplot2 v3.4.4 [22]. To calculate the historical *N_e_* and contemporary *N_e_* of the populations, the methodology described by Santiago et al. [23] was used with data from unphased diploid genotypes. For the calculation of historical *N_e_*, GONE2 software v2.0 was used, and the trajectory of *N_e_* was estimated up to 100 generations considering a constant recombination rate of 1 cM/Mb using the -r command. To calculate contemporary *N_e,_* the current Ne2 software v2.0 was used considering a sample of 50,000 SNPs and assuming a constant recombination rate of 1 cM/Mb.

### 2.5. Population Structure

To analyze the structure of the two Mexican Creole cattle populations, a merged dataset was constructed using genotypes of reference cattle populations obtained from the Web-Interfaced Next Generation Database dedicated to Genetic Diversity Exploration (WIDDE; available online: http://widde.toulouse.inra.fr/widde/, accessed on 30 January 2026) [24]. Animals (*Bos taurus*) of the Holstein (HOL; *n* = 56) and Angus (ANG; *n* = 42) breeds were included as representatives of the European taurine cattle, animals (*Bos taurus*) of the N’Dama (NDA; *n* = 23) breed were included as representatives of the African taurine cattle, and animals (*Bos indicus*) of the Gyr breed (GIR; *n* = 27) were included as representatives of indicine cattle. After merging, only shared autosomal SNPs were retained for downstream analyses. No additional filtering for missingness or minor allele frequency was applied to the merged dataset. To reduce bias associated with correlated markers, an LD filter was applied in PLINK v1.9 [17] using the --indep-pairwise command considering sliding windows of 50 SNPs, a step size of 5 SNPs, and an r^2^ threshold of 0.2. Highly correlated SNPs were removed until the established criterion was met. To analyze the population structure in an unsupervised manner, principal component analysis (PCA) was performed with PLINK v1.9 [17] using the command --pca. To visualize the position of each individual considering the coordinates of the first two principal components, a scatter plot was constructed using the ggplot2 library [22] of R software [21]. To analyze the differentiation between populations, a discriminant principal component analysis (DAPC) was performed using the adegenet v2.1.7 library [25] in R software [21]. The number of clusters and principal components to retain were calculated using the find.clusters and xvalDapc functions of the library. Additionally, the population structure was evaluated via an ancestry analysis using ADMIXTURE v1.3 [26], which uses a cross-validation procedure to determine the best K value in the populations, and a bar graph was constructed using the libraries ggplot2 v3.4.4 [22], dplyr v1.1.4 [27] and tidyr v1.3.1 [28] in R software [21].

## 3. Results

### 3.1. Genetic Diversity

After quality control, genotype data from both populations were combined, resulting in a final dataset of 74,731 autosomal SNPs and 75 animals. The genetic diversity indicators estimated for these populations are shown in Table 1. The CR population showed a higher MAF value (0.311), than the CC population (0.291). Observed heterozygosity (Ho) was similar in both populations (CC = 0.403; CR = 0.400), while the expected heterozygosity (He) was higher in CR (0.396) than in CC (0.380). Both populations showed negative Fis and F values, indicating a slight excess of heterozygotes, with this effect being greater in the CC population (Fis −0.061, F −0.040) than in the CR population (Fis −0.010, F −0.007). The genomic inbreeding coefficient estimated from runs of homozygosity (FROH) was higher in CC (0.048) than in CR (0.036). The estimated Fst value (0.040) indicated low genetic differentiation between the two populations.

The distribution of ROH segments by length classes is shown in Table 2. In the CC population, a total of 1397 ROH segments were identified, with an average length of 3.10 Mb (SD = 5.38; min = 0.50; max = 69.78). The short segments (>0.5–4) represented the greatest proportion of genomic coverage, contributing to 88.16% of the total segments. In the CR population, 1658 ROH segments were detected, with an average length of 2.10 Mb (SD = 1.83; min = 0.51; max = 24.76), and the short segments (>0.5–4) represented 94.21% of the total ROH segments. Compared with the CR population (>8 Mb; 1.33%), the CC population showed a greater proportion of long ROH segments (>8 Mb; 6.08%).

### 3.2. Linkage Disequilibrium

**Figure 1:**
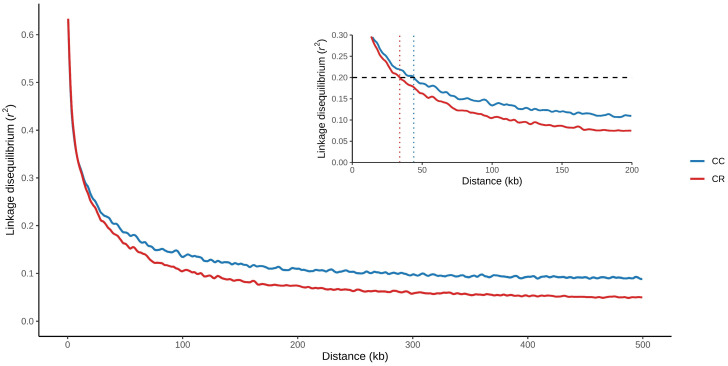
Linkage disequilibrium (LD) decay in two Mexican Creole cattle populations. Coreño (CC) and Raramuri (CR) are indicated. The horizontal dashed line indicates the r^2^ = 0.20 threshold, while the colored vertical dashed lines mark the distance at which LD decayed below this threshold in each population: 34 kb in CR and 43 kb in CC.

**Figure 2:**
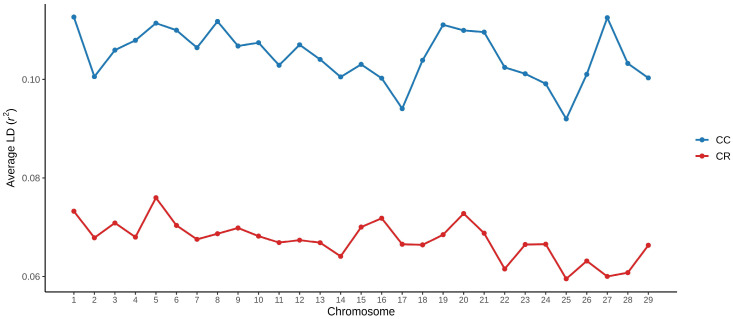
Average LD (r^2^) observed between the autosomal chromosomes of the Coreño (CC) and Raramuri (CR) populations.

According to Figure 1, the LD decay below the threshold of r^2^ = 0.20 was reached at a shorter distance in the CR population (34 Kb). In contrast, in the CC population, the LD extended for a longer distance (43 Kb). The LD exhibited heterogeneity between chromosomes in both populations. The average r^2^ per chromosome is shown in Figure 2. In general, the CC population exhibited a higher average LD (r^2^ = 0.1050 ± 0.0053) than that observed in the CR population (r^2^ = 0.0678 ± 0.0040). Despite the observed difference in magnitude, the results of the Spearman correlation analysis supported by permutations revealed moderate concordance of the chromosomal profile between populations in a positive sense (ρ = 0.539), as well as a significance with a value of *p* < 0.05 (*p* = 0.0030). In addition, the overlap of autosomes with extreme values was greater than expected by chance (*p* = 0.0209), identifying chromosomes with consistently high (BTA1, BTA5, BTA6 and BTA20) and low (BTA25, BTA26 and BTA29) r^2^ values in both populations, respectively.

**Figure 3:**
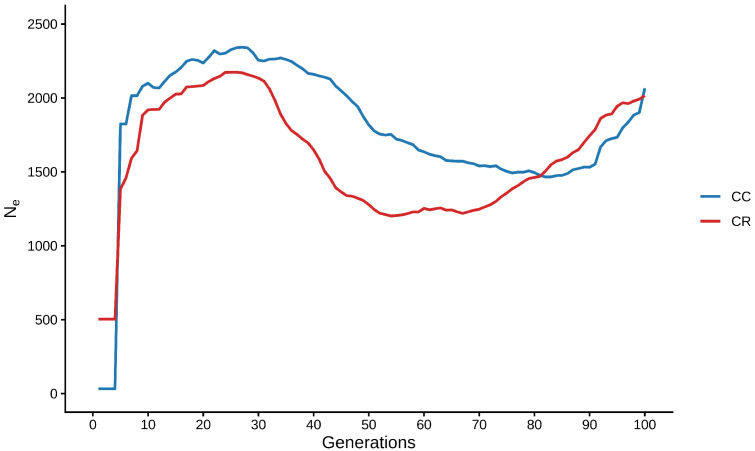
Historical *N_e_* determined using GONE2 considering the last 100 generations of the Coreño (CC) and Raramuri (CR) populations.

The historical effective size was estimated from LD up to 100 generations, which allowed us to reconstruct the demographic history of both populations. The data in Figure 3 reveal a slight reduction in *N_e_* in both populations from generation 100 to generation 70, followed by a sustained increase until the last 10 contemporary generations and then by a drastic reduction in effective size in both populations. The contemporary *N_e_* was 32 for the CC cattle and 473 in CR cattle, suggesting that the CC population may be at greater risk of genetic drift and loss of diversity.

### 3.3. Population Structure

**Figure 4:**
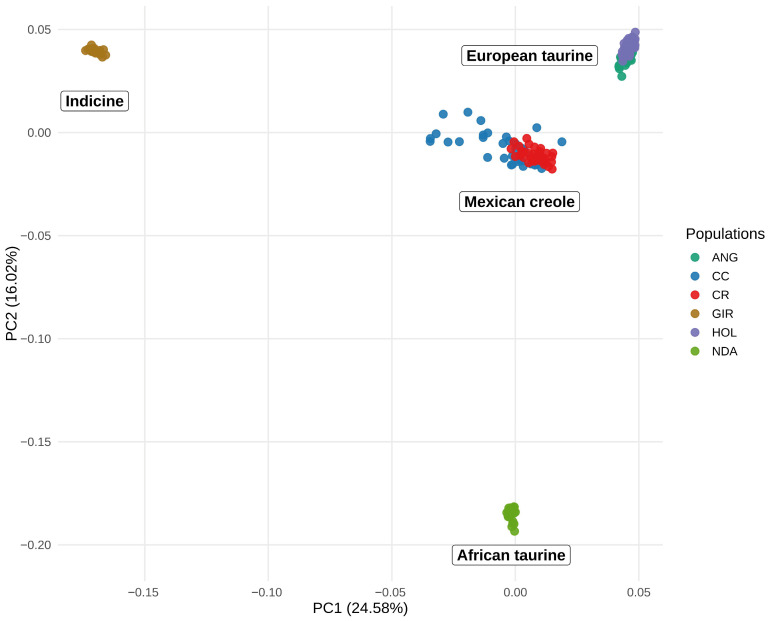
Principal component analysis (PCA) plot including the reference populations. The x- and y-axes represent principal components 1 and 2, respectively. The populations are labeled Angus (ANG), Holstein (HOL), Coreño (CC), Raramuri (CR), N’Dama (NDA) and Gyr (GIR).

PCA was performed to determine the population structure using a final dataset of 27,921 SNPs and 223 animals. As shown in the PCA plot in Figure 4, principal component 1 (PC1) represented 24.58% of the total variation and resulted in the separation of the populations of the subspecies *Bos taurus* and *Bos indicus*, whereas principal component 2 (PC2), which represented 16.02% of the total variation, resulted in the separation of the populations of taurine cattle into the European, Mexican and African groups. PCA revealed overlap of some animals in the CC and CR populations. The DAPC enabled the identification of 6 genetic groups in the analyzed populations, which was consistent with the allocation of the animals to their respective populations, with the exception of some animals of the CC population, which were assigned to the CR population, confirming the substructure of the Creole populations of Mexico.

**Figure 5:**
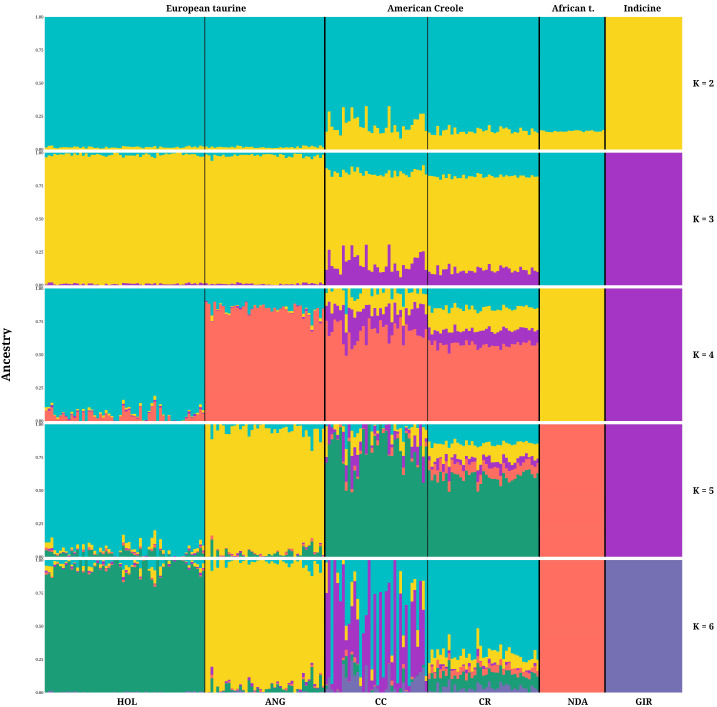
ADMIXTURE bar plots showing the inferred ancestry proportions for K = 2–6. Each vertical bar represents one individual, and colors indicate the estimated ancestry components. Individuals are grouped by population: Holstein (HOL), Angus (ANG), Coreño (CC), Raramuri (CR), N’Dama (NDA), and Gyr (GIR).

ADMIXTURE analysis was performed considering different K values (2–6) to analyze the ancestral composition of the Mexican Creole cattle populations in relation to the reference populations (Figure 5). Among the tested models, the cross-validation error was lowest at K = 6; therefore, this value was considered the best-supported solution within the explored range for representing the ancestral components of the populations. At K = 2, a separation of components consistent with the *Bos taurus* and *Bos indicus* lineages was observed. At intermediate values (K = 3–5), ancestry components more specific to breed and population progressively emerged. At K = 6, the reference populations (HOL, ANG, NDA and GIR) exhibited predominant population-specific ancestry patterns, whereas the Mexican Creole populations showed slightly more heterogeneous profiles, with the predominant Creole ancestral component representing 70.60% of the CR cattle. The CC cattle shared 38.67% of this Creole component but differed in the emergence of a differentiated ancestral component, which represented 46.44%.

## 4. Discussion

### 4.1. Genetic Diversity

The estimation of indicators of genetic diversity using high-density markers, such as SNP microarrays, has been established as a key tool for evaluating the conservation status and demographic health of local cattle populations [29]. An elevated MAF value suggests a balanced gene pool and, consequently, a lower proportion of rare alleles. In our study, the average MAF was slightly higher in the CR population (MAF = 0.311) than in the CC population (MAF = 0.291). Despite this slight difference, both values were higher than those reported for South African Angus (MAF = 0.21) and Holstein (MAF = 0.22) populations evaluated with the BovineSNP50 array [30], although differences in population origin and study design should be considered when making comparisons. The pattern observed in the Creole cattle populations of Mexico is consistent with that described in other Creole cattle populations of the Americas, where the highest representation of SNPs was found in categories with MAF ≥ 0.3 [31,32].

The observed heterozygosity was similar in the CC (Ho = 0.403) and CR (Ho = 0.400) populations, whereas the expected heterozygosity was lower (He = 0.380 and 0.396, respectively), indicating an excess of heterozygotes. This deviation (Ho > He) was consistently reflected in the inbreeding coefficients based on frequencies: negative FIS (CC = −0.061; CR = −0.010) and negative F values (CC = −0.040; CR = −0.007). These results reflect a slight deficit of homozygotes with respect to the expected values, with a more pronounced difference in the CC cattle than in the CR cattle. These observed patterns coincide with those reported in Creole cattle populations of Mexico genotyped by SNP microarrays [4,12,32]. Although the design of the genotyping panel may introduce bias in estimates of genetic diversity when they are applied to local populations for which they were not specifically developed [33]. Likewise, differences in quality control procedures across studies limit direct comparison of absolute values [34]. However, the available evidence indicates that intense artificial selection can contribute to erosion of genetic diversity as is observed in Holstein cattle, which have shown reduced heterozygosity [35] and increased inbreeding after the implementation of genomic selection [36].

With respect to the study of homozygosity, the FROH was similar in both Creole populations, representing 4.8% of the genome in a homozygous state in the CC cattle and 3.6% in the CR cattle. The heterogeneity of the parameters used to detect the ROH segments suggests that comparisons of results from different studies should be performed with caution [37]. Nevertheless, the values of our study are consistent with those reported for other Creole populations in America (FROH = ~0.03–0.05) [38]. In contrast to these results, in some Creole populations maintained in conservation nuclei with limited gene flow, higher FROH values have been observed, such as in Criollo Lechero Tropical (FROH = 10.9 ± 3.0%) and Romosinuano (FROH = 7.28 ± 3.68%) in Mexico [39,40]. According to the ROH length classes analyzed, short segments (0.5–4 Mb) accounted for the largest proportion of the genome covered by ROH in both populations. This pattern suggests that a substantial fraction of the detected homozygosity is associated with ancient inbreeding processes, as previously proposed for Raramuri cattle [4].

In comparison, the CR population exhibited a more stable genomic profile, with greater intrapopulation diversity, as reflected by MAF and slightly higher expected heterozygosity. The similarity between Ho and He, together with the lower FROH and reduced contribution of long ROH, suggests that most of the detected homozygosity in this population is linked with older demographic processes rather than to recent inbreeding. Thus, despite the geographic isolation of the region where CR cattle are raised, the genomic evidence does not support a stronger signal of recent mating among related individuals in this population.

In contrast, the CC population exhibited signals compatible with greater recent homozygosity, as evidenced by a greater contribution of long ROH (>8 Mb) and the slightly higher FROH, which is consistent with more extended LD and a smaller contemporary effective population size [36]. Because long ROH are generally associated with more recent common ancestry, this pattern suggests that mating among related individuals may have been more frequent in CC in recent generations. Although direct historical records of herd management are not available in the present study, biologically plausible explanations include a more restricted recent breeding pool, fragmentation into relatively small management groups, limited exchange of breeders, or the disproportionate use of a few sires. At the same time, the greater deviation of Ho with respect to He, reflecting an excess of heterozygotes, indicates that the overall heterozygosity and the homozygosity detected by ROH analysis may represent different and non-exclusive components of the demographic history of the population, as has been observed in other studies of populations in which relatively high global genetic diversity coexists with recent homozygosity due to effective size reduction and admixture or introgression [41].

Taken together, the diversity and homozygosity patterns observed in both populations indicate the retention of valuable genomic variation, while also suggesting a less favorable recent demographic scenario in CC than in CR. These findings support the relevance of Mexican Creole cattle as reservoirs of diversity with potential importance for adaptation, resilience and conservation.

### 4.2. Linkage Disequilibrium

Owing to the cumulative effect of recombination, LD progressively decreases as the physical distance between SNPs increases [42]. A common approximation to describe the extent of LD consists of reporting the distance at which the value of r^2^ falls below a reference threshold. In our study, the decay in the CR population was reached at a shorter distance (34 Kb) when the threshold of r^2^ = 0.2 was considered. In the CC cattle, the threshold was reached at a longer distance (43 kb), indicating a greater extent of nonrandom associations between markers throughout the genome. The magnitude and extent of LD should be interpreted with caution when comparisons between populations are being made [43], as its behavior responds to historical demographic events in each population and may reflect processes such as genetic flow, mixing, selection or a decrease in *N_e_*. This has been reported in Blanco Orejinegro Creole cattle populations [31]. In cattle of the species *Bos taurus* selected for meat production, the value of r^2^ decreased below 0.2 at approximately 50 Kb [44]. In intensely selected breeds of cattle, a slower declining pattern was observed compared with local populations with less intervention, which usually retain greater genetic diversity and lower levels of linkage between loci [45].

With respect to the heterogeneous behavior of LD across autosomes, chromosomes with consistently low LD values were identified (BTA25, BTA26 and BTA29), which may reflect greater historical recombination and breakdown of allelic associations. In contrast, autosomes with consistently high LD (BTA1, BTA5, BTA6 and BTA20) were also observed, which could be associated with less effective recombination and with demographic or selective processes affecting specific genomic regions, as has been reported in other Creole cattle populations [46].

Considering the generation interval of approximately 5–6 years reported for some Creole cattle populations in the Americas [47,48], the estimation of historical *N_e_* over the last 100 generations allowed us to explore demographic changes in Mexican Creole cattle since their introduction into the continent more than 500 years ago. In this context, LD-based inference of *N_e_* provides a useful framework for reconstructing the demographic history of these populations.

As in our study, previous reports suggest that the demographic trajectory of Creole cattle in the Americas can be broadly summarized in three stages. The first corresponds to a foundation phase from a small number of animals, consistent with an initial bottleneck that likely reduced genetic diversity, extended LD, and decreased *N_e_*. The second stage was characterized by population expansion and geographical dispersion during colonization, which likely increased *N_e_*. The third stage corresponds to the contemporary contraction, in which *N_e_* declines markedly, probably due to the replacement of native animals by commercial breeds, introgression from foreign germplasm and additional bottleneck events [38,39,49].

With respect to this marked decrease in contemporary *N_e_*, the CC population showed a value of 32, which is similar to those reported for other Creole populations such as Raramuri cattle (*N_e_* = 33), Criollo Lechero Tropical (*N_e_* = 56), Peruvian Creole (*N_e_* = 36.4), Argentine Creole (*N_e_* = 9.6) and Uruguayan Creole (*N_e_* = 13.8) [4,12,38]. This value is of particular concern because it falls below the threshold of 50 which is widely cited as a minimum reference for the short-term conservation of genetic diversity [50]. Although LD-based estimates of contemporary *N_e_* may be affected by sample size and population structure, the low value observed in CC is consistent with the broader genomic patterns detected in this population, including slower LD decay, a greater contribution of long ROH segments, and a slightly higher FROH. Moreover, the recent demographic trend inferred with GONE2 was consistent with recent effective size reduction in CC, which supports the biological plausibility of the estimate obtained with currentNe2.

In contrast, the CR population exhibited a greater *N_e_* (*N_e_* = 473), indicating greater demographic stability and better retention of genetic diversity over time. Together, these results suggest that CC cattle may face greater genetic vulnerability and should therefore be prioritized in conservation planning. In practical terms, conservation efforts for this population should focus on limiting further loss of diversity by using genomic information to identify less-related animals, avoid the disproportionate use of a few breeders, and support mating schemes that reduce recent inbreeding while retaining as much of the existing genomic variation as possible. Continued genomic monitoring would also be valuable to assess changes in diversity, homozygosity, and effective population size over time.

### 4.3. Population Structure

The characterization of population genetic structure is essential for the conservation and management of animal genetic resources because it helps identify distinctive genetic components, detect admixture signals in local populations, and guide breeding strategies aimed at maintaining population integrity [51]. To evaluate the genomic composition of Mexican Creole cattle and to assess the relative influence of European, African and indicine ancestral components, we included reference populations representative of these major cattle lineages.

The PCA clearly separated populations according to the broad genetic groups *Bos taurus* and *Bos indicus*, and further distinguished European, African and American-derived backgrounds. Within this framework, both Mexican Creole populations were positioned closer to European taurine cattle. This result is consistent with the documented historical origin of Creole cattle in the Americas, which traces back primarily to Iberian cattle introduced during the colonial period [1,4,12]. Therefore, the proximity of CC and CR to European populations likely reflects the persistence of this shared historical foundation rather than recent admixture with modern commercial breeds alone.

The overlap between CC and CR in the PCA, together with the low pairwise Fst and the DAPC results, indicates limited genetic differentiation between these two populations. This pattern suggests that, despite their current geographic and management separation, both populations still retain a substantial proportion of a common ancestral genomic background. In biological terms, the low differentiation observed here is compatible with descent from related founder stocks, followed by divergence that has been insufficient to generate strong genomic separation. Such a pattern is expected in populations derived from a common Creole background, particularly when differentiation has occurred under moderate drift and local adaptation rather than under long-term reproductive isolation [1].

The ADMIXTURE results were concordant with the multivariate analyses. At lower K values (K = 2–5) the models captured the broad ancestral partitioning among major cattle groups, whereas at K = 6, the structure of the analyzed populations was represented in greater detail. Under this model, the CR population was characterized predominantly by a Creole ancestral component, while CC shared this same ancestral component but also showed a more noticeable contribution of an additional differentiated ancestry signal in some individuals. This pattern suggests that CC is genetically more heterogeneous than CR.

The broader dispersion of CC individuals in the PCA and the more complex ancestry profile observed in ADMIXTURE may reflect several non-mutually exclusive processes. One possibility is stronger internal subdivision within CC, potentially associated with breeding in partially isolated herds or subpopulations. Another is a more heterogeneous history of gene flow or introgression, which could have introduced differentiated genomic segments into some animals [1,51]. These scenarios are also consistent with the diversity results, which indicated greater dispersion in some genomic parameters and a stronger signal of recent homozygosity in CC. Thus, the observed population structure does not merely describe clustering patterns, but also suggests a more complex recent demographic history in CC than in CR.

From a conservation perspective, these results are particularly relevant. The predominance of a shared Creole component in both populations supports their value as reservoirs of Mexican Creole genomic heritage, whereas the greater heterogeneity detected in CC suggests that this population may require more careful monitoring and management. Conservation strategies for CC should aim to preserve as much of its existing genomic variation as possible while avoiding further loss of diversity through the disproportionate use of a limited number of breeders. In contrast, the more homogeneous ancestry profile observed in CR may reflect greater genomic cohesion, although this should not be interpreted as absence of conservation concern.

A limitation of this study is the relatively small number of animals analyzed, which should be taken into account when interpreting the results. This may influence, to some extent, the precision of some population-level estimates, particularly those related to genetic diversity, ROH patterns, LD, and effective population size. Therefore, the findings should be considered as an initial genomic characterization of the sampled CC and CR animals. Future studies with broader sampling across herds, regions, and generations will be necessary to validate these patterns and provide more precise estimates to support conservation planning. Despite this limitation, the consistency among the different genomic analyses strengthens the overall interpretation and emphasizes the value of genomic information as a complementary tool for conservation planning in Mexican Creole cattle.

## 5. Conclusions

In this study, the genetic diversity and population structure of two Mexican Creole cattle populations were characterized, providing genomic evidence of the relevance of these populations as local genetic resources for conservation and sustainable management. The analyses showed substantial genetic diversity within both Creole cattle populations, low genomic FROH values, and a predominance of short ROH segments of the 0.5–4 Mb class, suggesting that recent inbreeding is not severe in either population. LD analysis revealed a greater extent of linkage disequilibrium in the CC population, which was consistent with its lower contemporary effective population size and suggests the need to prioritize conservation strategies aimed at maintaining genetic diversity, minimizing future inbreeding, and guiding planned mating schemes. Population structure analyses indicated a closer relationship of the Creole cattle with European taurine populations, low genetic differentiation between the Creole populations, and ancestry patterns characterized by the predominance of a Creole ancestral component in the CR population, whereas CC cattle shared this component and exhibited an additional differentiated ancestral component. These findings indicate that both populations retain valuable genetic variation but also show population-specific genomic patterns that should be considered when designing conservation and breeding strategies. Overall, the consistency among diversity, ROH, LD, effective population size, and population structure analyses provides useful information to support future conservation programs for Mexican Creole cattle.

## Figures and Tables

**Table 1:** Summary of genetic diversity indicators in two Mexican Creole cattle populations.

POP	*n*	MAF ± SD	Ho ± SD	He ± SD	Fis ± SD	F ± SD	FROH ± SD	Fst
CC	36	0.291 ± 0.135	0.403 ± 0.023	0.380 ± 0.0001	−0.061 ± 0.0619	−0.040 ± 0.0398	0.048 ± 0.0471	0.040
CR	39	0.311 ± 0.128	0.400 ± 0.009	0.396 ± 0.0000	−0.010 ± 0.0219	−0.007 ± 0.0144	0.036 ± 0.0153	
*p*-value		8.59 × 10^−179^	0.205	9.90 × 10^−14^	1.22 × 10^−6^	1.51 × 10^−6^	0.987	

Values are presented as mean ± SD. *p*-values correspond to the comparison between CC and CR using the Mann–Whitney U test. Fst is presented as a descriptive estimate of genetic differentiation between populations. SD = standard deviation.

**Table 2:** Distribution of ROH by length class in two Mexican Creole cattle populations.

POP	Class (Mb)	Number	%	FROH Mean	SD	Range
CC	>0.5–1	95	6.8	0.00091	0.00051	0.00036–0.00204
	>1–2	753	53.9	0.01216	0.00581	0.00173–0.02245
	>2–4	384	27.49	0.01129	0.00676	0.00082–0.02525
	>4–8	80	5.73	0.00474	0.00629	0.00162–0.02897
	>8–16	40	2.86	0.00507	0.01057	0.00345–0.03943
	>16	45	3.22	0.01422	0.03260	0.01303–0.12998
CR	>0.5–1	113	6.82	0.00097	0.00055	0.00028–0.00277
	>1–2	987	59.53	0.01487	0.00401	0.00761–0.02448
	>2–4	462	27.86	0.01262	0.0041	0.00281–0.01969
	>4–8	74	4.46	0.00403	0.0035	0.00161–0.01464
	>8–16	16	0.97	0.00208	0.0062	0.00372–0.03195
	>16	6	0.36	0.00123	0.0048	0.00656–0.02449

## Data Availability

The data generated in this study is not available because it is part of an ongoing investigation.
